# The complete mitochondrial genome of yellowfin seabream, *Acanthopagrus latus* (Percoiformes, Sparidae) from Beibu Bay

**DOI:** 10.1080/23802359.2021.1907804

**Published:** 2021-03-31

**Authors:** Chuanyan Pan, Chongmin Gao, Tao Chen, Xiuli Chen, Chunling Yang, Digang Zeng, Pengfei Feng, Weiming Jiang, Min Peng

**Affiliations:** a Guangxi Key Laboratory of Aquatic Genetic Breeding and Healthy Aquaculture, Guangxi Academy of Fishery Sciences, Nanning, China; b Guangxi Agricultural Vocational College, Nanning, China

**Keywords:** Yellowfin seabream, *Acanthopagrus latus*, mitochondrial genome, Sparidae

## Abstract

The yellowfin seabream, *Acanthopagrus latus* Houttuyn 1782, is a commercially and ecologically important species and a good model for studies of sexual differentiation. In this study, the complete mitochondrial genome of *A. latus* has been determined, which is 16,635 base pairs (54.3% A + T content) in length and consists of 13 protein-coding genes, 22 transfer RNAs, two ribosomal RNAs, and a 948 bp D-loop region. The phylogenetic analyses showed that *A. latus* has a close relationship with *Acanthopagrus schlegelii* Bleeker 1854.

The yellowfin seabream (*Acanthopagrus latus*) is a commercially and ecologically important species, which is a popular food in Asia due to its beautiful appearance, delicious taste, and nutritional value (Xia et al. 2008; Li et al. 2020). *A. latus* is widely distributed throughout the Indo-West Pacific (Xia et al. 2008), and it usually inhabits warm shallow and coastal waters. Wild resources of *A. latus* have severely decreased because of overfishing and habitat loss. *A. latus* is a good model for studying sexual differentiation due to its characteristics of protandrous hermaphroditic. Although phylogenetic analyses of Sparidae have been performed, the phylogenetic position of its members has not yet been determined (Orrell and Carpenter 2004; Xia et al. 2008). Mitochondrial DNA (mtDNA) is an important model system for studying molecular evolution and phylogeny because of its characteristics of constant gene content, maternal inheritance, lacking of recombination and paralogous genes (Podsiadlowski et al. 2007). The complete mitochondrial genome is an excellent molecular marker for studying phylogenetic relationships and species identification (Zhong et al. 2020). Here, we report the complete mitochondrial genome of *A. latus* and its genetic distance between sixteen other species. The results of this study will provide reference for genetics research of *A. latus*.

The *A. latus* samples were collected from Weizhou island (21.027201 N, 109.133285E), Beihai city, Guangxi province, China. The whole body specimens (specimens number: #BH201905310002) were deposited at Guangxi key laboratory of aquatic genetic breeding and healthy aquaculture. The total genomic DNA was extracted from the fins of one *A. latus* sample via the phenolchloroform extraction method (Kumar and Mugunthan 2018). DNA libraries were constructed using the TruSeq NanoTM kit (Illumina, San Diego, CA) and sequenced (2 × 150 bp paired-end) on a HiSeq platform by Novogene Company, China. Sequence assembly was performed using the MITObim software (Hahn et al. 2013). Gene annotation was performed using the MITOS software (http://mitos2.bioinf.uni-leipzig.de/).

The complete mitochondrial genome of *A. latus* is 16,635 bp in length (GenBank accession number: MN909968) with the base composition of A (24.4%), T (30.1%), C (29.4%), and G (16.3%). The percentage of G + C is 45.7% and the percentage of A + T is 54.3%, which is similar to the *A. latus* collected from Guangdong (Xia et al. 2008) and *Acanthopagrus schlegelii* (44.1% of G + C and 55.9% of A + T) (Shi et al. 2012). The mitochondrial genome contains 13 protein-coding genes (11,443 bp), 22 transfer RNAs (with a range in size from 67 to 74 bp), and two ribosomal RNAs (a 12S rRNA and a 16S rRNA), and one 948 bp control region locates between the tRNA-Pro gene and tRNA-Phe gene. In the complete mitochondrial genome of *A. latus* collected from Guangdong province, the total size of 13 protein-coding genes was 11,422 bp, the size range of transfer RNAs was 68–75 bp, the control region was 943 bp, and the percentages of A + T and G + C are 55.2% and 44.8%, respectively (Xia et al. 2008).

All the 13 protein-coding genes were concatenated into a single sequence for phylogenetic analysis using the Bayesian inference technique. The result showed that *A. latus* was first clustered with *A. latus* from Guangdong (EF 506764.1) and then clustered with *Acanthopagrus schlegelii* (*A. schlegelii*) (Figure 1). The complete mitochondrial genome sequence of *A. latus* will enrich the genome data of Sparidae and will be useful for taxonomy research, conservation and management.

**Figure 1.**
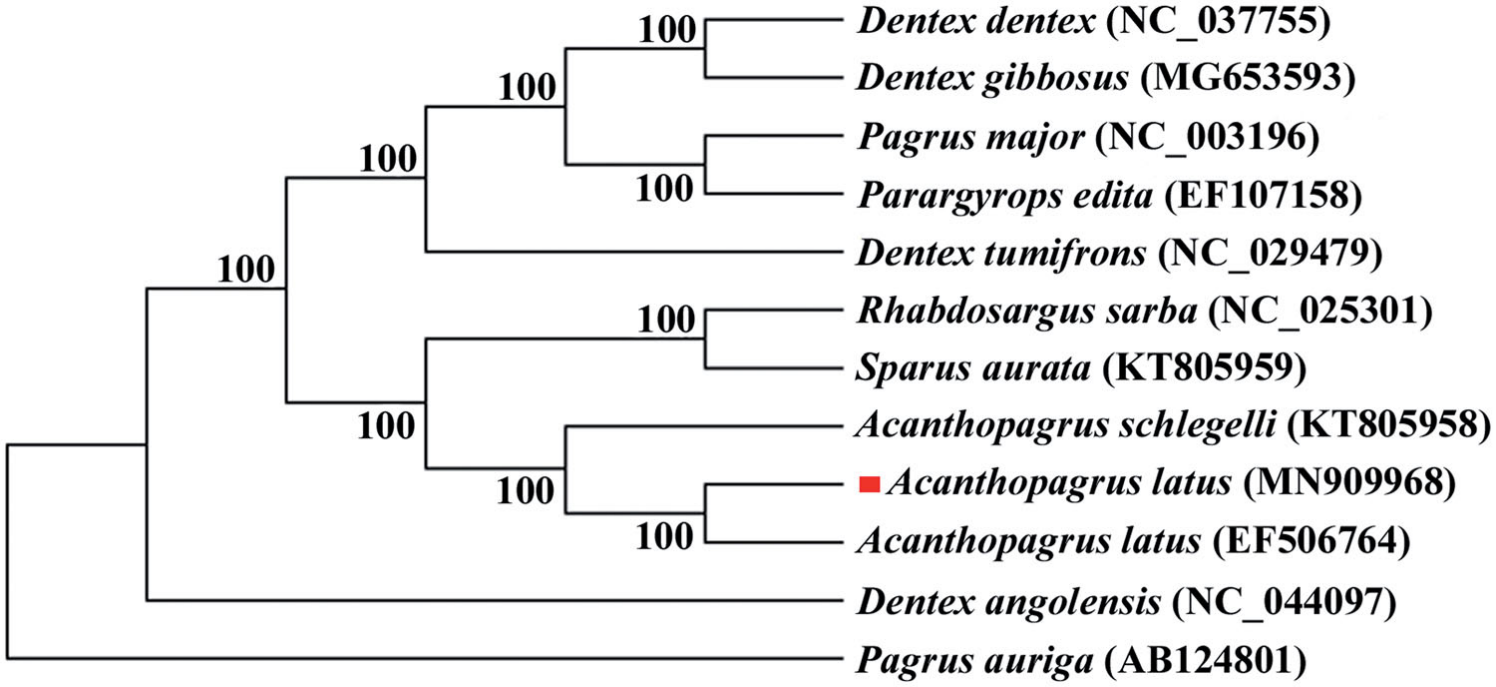
A phylogenetic tree was constructed based on the comparison of mitochondrial genome sequences of A. latus and other species. All the sequences were downloaded from NCBI GenBank.

## Data Availability

The genome sequence data that support the findings of this study are openly available in GenBank of NCBI at (https://www.ncbi.nlm.nih.gov/) under the accession nomber [MN909968]. The associated BioProject, SRA, and BioSample numbers are PRJNA688781, SRR13334792, and SAMN17188822, respectively.
